# Change in Breast Cancer Screening Participation during COVID-19 Based on the 2019 and 2022 Comprehensive Survey of Living Conditions in Japan

**DOI:** 10.31662/jmaj.2024-0234

**Published:** 2024-12-20

**Authors:** Chitose Kawamura, Masao Iwagami, Jun Komiyama, Yuta Taniguchi, Yu Sun, Rie Masuda, Takehiro Sugiyama, Hiroko Bando, Tomomi Kihara, Hiroyasu Iso, Nanako Tamiya

**Affiliations:** 1 Department of Health Services Research, Graduate School of Comprehensive Human Sciences, University of Tsukuba, Ibaraki, Japan; 2 Department of Breast-Thyroid-Endocrine Surgery, University of Tsukuba Hospital, University of Tsukuba, Ibaraki, Japan; 3 Department of Health Services Research, Institute of Medicine, University of Tsukuba, Ibaraki, Japan; 4 Health Services Research and Development Center, University of Tsukuba, Ibaraki, Japan; 5 Institute for Global Health Policy Research, Bureau of International Health Cooperation, National Center for Global Health and Medicine, Tokyo, Japan; 6 Higashigaoka Faculty of Nursing, Tokyo Healthcare University, Tokyo, Japan; 7 Diabetes and Metabolism Information Center, Research Institute, National Center for Global Health and Medicine, Tokyo, Japan; 8 Department of Breast and Endocrine Surgery, Institute of Medicine, University of Tsukuba, Ibaraki, Japan *Co-last authors; 9 Co-last authors

**Keywords:** breast cancer, cancer screening, COVID-19, pandemic, Japan

## Abstract

**Introduction:**

The breast cancer screening rate declined worldwide during the COVID-19 pandemic. This cross-sectional study examined the changes in breast cancer screening participation rates in Japan before and during the pandemic and identified subgroups with a larger decline.

**Methods:**

We used data from a 2019 survey evaluating 2017-2018 (pre-pandemic) and a 2022 survey evaluating 2020-2021 (during the pandemic) in the Comprehensive Survey of Living Conditions to describe the breast cancer screening rates by screening settings among women aged 40-74 years. We calculated the changes in the overall participation rate and by subgroup with and without adjustment for other variables (i.e., age, living area, educational level, and health insurance).

**Results:**

The participation rates in breast cancer screening in 2017-2018 and 2020-2021 were 48.3% (51,428/106,446, municipality-based 18.7%, worksite-based 17.0%, and others 12.6%) and 47.1% (45,006/95,610, municipality-based 17.2%, worksite-based 17.5%, and others 12.4%), respectively. The crude difference from 2017-2018 to 2020-2021 was −1.2% (95% confidence interval [CI], −1.7 to −0.8), and the adjusted difference was −1.7% (−2.2 to −1.4). By subgroup, the adjusted difference was the largest in the 45-49 age subgroup (−2.2% [−3.3 to −1.1]) among the age subgroups, in the town/village subgroup (−2.4% [−3.6 to −1.2]) among the living area subgroups, in the high school subgroup (−1.8% [−2.4 to −1.2]) and vocational school/junior or technical college subgroup (−1.8% [−2.6 to −1.0]) among the educational level subgroups, and in the employee insurance (dependent person) subgroup (−2.5% [−3.3 to −1.7]) among the health insurance subgroups.

**Conclusions:**

The breast cancer screening participation rates decreased during the pandemic in Japan, with some variations by subgroup. For the screening setting, the participation rate of the municipality-based screening decreased, while that of the worksite-based screening increased.

## Introduction

The COVID-19 pandemic induced behavioral changes globally. In Japan, a nationwide declaration of a state of emergency was issued for the first time in April 2020. Subsequently, the declaration of the state of emergency was issued thrice during the period from January 2021 to September 2021, spanning over approximately 6 months mainly in populated areas ^(1)^. During this time, people were advised to avoid non-essential and non-urgent outings.

Globally, breast cancer screening rates declined because of the pandemic ^(2), (3)^. According to overseas studies, the breast cancer screening rates and the number of breast cancer screenings sharply decreased in the early stages of the pandemic, and then recovered in the later stage, but did not return to their pre-pandemic level ^(4), (5), (6)^. Studies outside of Japan identified factors, such as low socioeconomic status, immigrant status, rural residence, and absence of a family doctor, as contributors to the breast cancer screening disparities during the pandemic ^(7), (8), (9)^.

In Japan, a study examining the number of people who underwent breast cancer screening found that the number of municipality-based breast cancer screenings substantially decreased in 2020 compared to those in 2017-2019 ^(10)^. Another study comparing the breast cancer screening rates before and after the pandemic reported that the proportion of people who regularly participated in screening (every 2 years as recommended) before the pandemic was 38.2%. For the last 2 years of the pandemic, the proportion of those who regularly or irregularly participated in screening became 46.9% ^(11)^. However, the former study only compared the number of municipal-based screenings, while the latter used different definitions to measure the participation rates before and during the pandemic (regular participation rate vs regular/irregular participation rate). Therefore, whether there was any nationwide change in the breast cancer screening rate before and during the pandemic in Japan remained unclear.

The Comprehensive Survey of Living Conditions (CSLC) is used in the Organization for Economic Cooperation and Development health statistics as the official data for the breast cancer screening rate in Japan ^(12)^. The CSLC evaluates the proportion of people who regularly or irregularly participated in breast cancer screening. The CSLC covers municipality- and worksite-based screenings and other opportunities ^(13)^, such as Ningen Dock, where individuals participate voluntarily and cover the full cost out-of-pocket ^(14)^. The characteristics of screening participants (e.g., health insurance), screening costs, and degree of recommendation for screening differ among municipal- and workplace-based screenings and Ningen Dock; hence, it is important to use CSLC to assess the breast cancer screening participation rate in Japan. Using the CSLC, we aimed to examine the changes in the breast cancer screening participation rates before and during the pandemic considering a consistent definition of the screening rate and identify subgroups with a larger decline in the participation rates in Japan.

## Materials and Methods

### Data source

The CSLC is a nationwide cross-sectional survey of people living in Japan comprising a self-report questionnaire conducted from Japan’s Ministry of Health, Labour and Welfare (MHLW). Since 1986, large-scale surveys, including household, health, income and savings, and long-term care surveys, have been conducted every 3 years. All household members are eligible for the survey, except for those absent from the household for some reason during the survey period (e.g., business travelers and students absent for more than 3 months) ^(15)^. We used the household and health data from the 2019 and 2022 surveys, which included approximately 280,000 households within 5,500 randomly selected stratified census tracts. The eligible households differed among the 2019 and 2022 surveys. The valid response rates for the questionnaires were 72.1% and 68.0% in 2019 and 2022, respectively.

The household questionnaire was divided into two parts: one was answered by one person in the household (five questions), and the other was answered by each household member (18 questions; the number of questions varies depending on age). The household questionnaire included age, educational level, and health insurance status. In the health questionnaire, each household member answered a series of questions (17 questions, the number of questions varied depending on age). The health questionnaire included questions about illnesses, drinking and smoking habits, and participation in the five cancer screenings (i.e., stomach, lung, colorectal, cervical, and breast). The detailed questions in the household and health questionnaires were listed in the online resources of our previous study ^(15)^. Regarding the study methods, the surveyor distributed the survey questionnaire in advance. The household member filled it in themselves, and the surveyor collected it later, or the household member responded using the online survey system of the government statistics joint use systems. For households encountering difficulty answering in person or online, it was also acceptable to send the answers by post ^(16)^. Those who wished to use the CSLC data must confirm meeting the relevant terms of use and apply to the MHLW ^(17)^.

We obtained approval from the MHLW to use the CSLC data. This study was approved by the Ethics Committee of the University of Tsukuba, Ibaraki, Japan under approval number 1754-1. The informed consent requirement was waived because anonymized data were obtained from the MHLW.

### Study population

We identified women aged 40-74 years based on the MHLW’s target age for individual screening recommendations at 40-69 years and the Japanese Breast Cancer Society’s optimal upper age range at approximately 75 years. We excluded ineligible women (hospitalized or in a long-term care facility) or women who did not respond to the question, “Have you participated in breast cancer screening (mammography or breast ultrasound) in the past 2 years?” and those who did not respond to questions regarding covariate variables.

### Outcome definition

The outcome variable was breast cancer screening participation over the past 2 years. The 2019 and 2022 surveys assessed the participation rates for 2017-2018 and 2020-2021, respectively. The women who responded to have participated in breast cancer screening also responded to the question about the screening setting (municipal- and workplace-based screening or other).

### Covariate (subgroup) definition

As in a previous study using the CSLC ^(15)^, we considered age, living area, educational level, and health insurance as the covariates potentially influencing the changes in breast cancer screening participation during the pandemic. We selected these covariates that seemed to modify the change in the participation rate between 2019 and 2022 (i.e., effect modifiers). These variables were unlikely to be affected by the COVID-19 pandemic.

Age was categorized into seven groups: 40-44, 45-49, 50-54, 55-59, 60-64, 65-69, and 70-74 years, following the National Cancer Center Japan’s classification ^(18)^. The living areas were categorized based on the population size: city with a population of ≥500 thousand people (government-designated cities or Tokyo’s 23 cities), city with 150-499 thousand people, city with 50-149 thousand people, city with <50 thousand people, and towns or villages. The educational level was categorized as elementary or junior high school, high school, vocational school or junior or technical college, and university or graduate school. The health insurance plan was categorized as employee insurance (insured person), employee insurance (dependent person), and national health insurance.

### Statistical analysis

We calculated the breast cancer screening participation rates in 2017-2018 and 2020-2021 and their crude differences overall and by the aforementioned variables, which were all categorical. Following the approach by Norton et al. ^(19)^, who employed logit models, we calculated the unadjusted and adjusted risk differences (i.e., the risk difference, adjusted for the difference of other variables between 2017-2018 and 2020-2021). The “adjrr” command was used to compute the adjusted risk ratio (ARR) and the adjusted risk difference (ARD). Norton et al. state the following:

“*The ARR and ARD are two ways to express the relationship between two predicted probabilities based on the fit model and a set of observations. One is the predicted probability when the variable of interest equals 1; the other is the predicted probability when the variable of interest equals 0 (more generally, pick any two values of the variable). These predicted probabilities are then averaged over the entire dataset (or perhaps an interesting subset of the data). The ARR is the ratio of the mean predicted probabilities, and the ARD is the difference of the mean predicted probabilities. The ARD is sometimes called the average treatment effect because it compares the effect of a change in the variable of interest (the treatment) for all observations*” ^(19)^.

For example, the ARD in the subgroup of women aged 40-44 means the risk difference if other variables (i.e., living areas, educational level, and health insurance) were equally distributed between 2017-2018 and 2020-2021 in this age group. We conducted a complete case analysis to exclude individuals with missing data. We did not conduct multiple imputations because missing data are likely to be missing not at random. We assumed no interaction between the variables. Furthermore, from 2021, the MHLW set the target age for active recommendation to 40-69 years old; therefore, we also calculated the breast cancer screening participation rate for people in this age range.

As a sensitivity analysis, because the survey response rate decreased from 72.1% in 2017-2018 to 68.0% in 2020-2021, consequently resulting in the decrease in the number of analyzed people from 106,446 in 2017-2018 and 95,610 in 2020-2021 (i.e., decrease of 10,836), we simulated the study results if the 10,836 people were more (up to 10%) or less (up to −10%) likely to participate in the breast cancer screening than the observed percentage (i.e., 47.1% in 2020-2021, as shown later).

All analyses were performed using STATA version 15.1 (Stata Corp, College Station, TX, USA).

## Results

**Figure 1.**
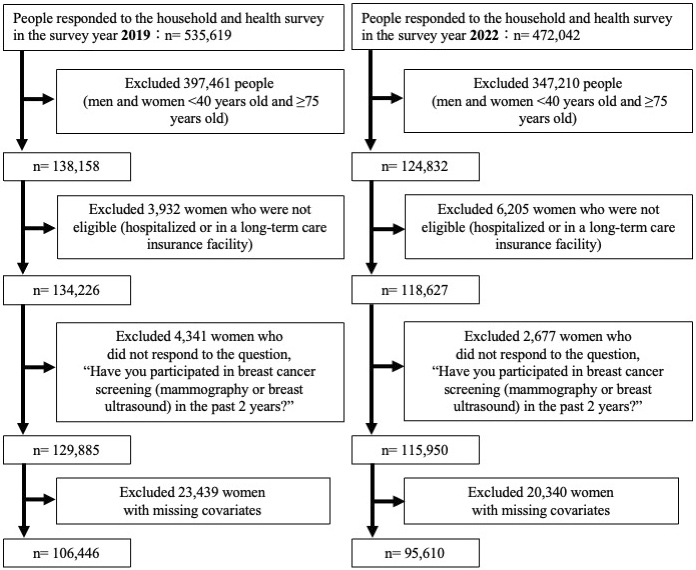
Flow chart of the study population selection in the 2019 (evaluating 2017-2018) and 2022 (evaluating 2020-2021) Comprehensive Survey of Living Conditions.

**Figure 2.**
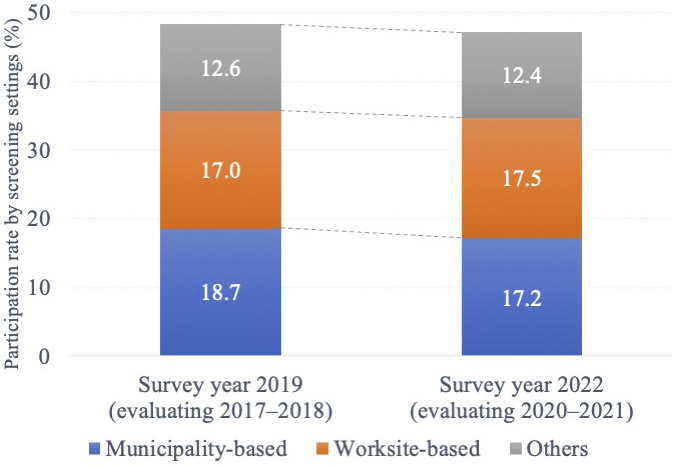
Participation rate of breast cancer screening by screening settings. Note: In the survey, multiple choices were possible, including municipality- and worksite-based screenings and other settings. If a respondent chooses two or more answers, we allocate them to the other group, such that every respondent is allocated to either municipality- or worksite-based or others in the figure.

Among the 535,619 and 472,042 CSLC participants in 2017-2018 and 2020-2021, respectively, we identified 138,158 and 124,832 women aged 40-74 years (Figure 1). After excluding those with missing data, 106,446 and 95,610 women were included in the subsequent analyses in 2017-2018 and 2020-2021, respectively. Table 1 shows the baseline characteristics and both the crude and adjusted differences in the breast cancer screening participation. The participation rates in breast cancer screening in 2017-2018 and 2020-2021 were 48.3% (51,428/106,446) and 47.1% (45,006/95,610), respectively, with the crude change from −1.2% (95% CI, −1.7 to −0.8). Regarding the breast cancer screening setting among participants, from 2017-2018 to 2020-2021, the municipality-based screening decreased from 18.7% to 17.2% with a crude difference of −1.5% (−1.8 to −1.1), while the worksite-based screening increased from 17.0% to 17.5% with a crude difference of 0.5% (0.2 to 0.9) (Figure 2).

**Table 1.** Baseline Characteristics of the Participants in the 2019 (Evaluating 2017-2018) and 2022 (Evaluating 2020-2021) Comprehensive Survey of Living Conditions and the Crude and Adjusted Differences in the Percentages of Participants Who Underwent Breast Cancer Screening.

	Participation rate in the survey year 2019 (evaluating 2017-2018)	Participation rate in the survey year 2022 (evaluating 2020-2021)	Crude difference from the survey year 2019 to the survey year 2022 (95% CI)		Adjusted difference from the survey year 2019 to the survey year 2022 (95% CI)	
Variables	% (participants/denominator)	% (participants/denominator)	%	p-Value	%	p-Value
Overall	48.3 (51,428/106,446)	47.1 (45,006/95,610)	−1.2 (−1.7 to −0.8)	<0.001	−1.7 (−2.2 to −1.4)	<0.001
Age category (years)						
40-44	55.1 (8,072/14,646)	54.4 (6,088/11,196)	−0.7 (−2.0 to 0.5)	0.238	−1.2 (−2.3 to 0.1)	0.061
45-49	55.2 (8,786/15,925)	54.2 (7,455/13,766)	−1.0 (−2.2 to 0.1)	0.080	−2.2 (−3.3 to −1.1)	<0.001
50-54	55.1 (8,073/14,657)	53.6 (7,416/13,838)	−1.5 (−2.7 to 0.3)	0.012	−1.9 (−3.1 to −0.8)	<0.001
55-59	52.5 (7,666/14,592)	52.0 (6,709/12,903)	−0.5 (−1.7 to 0.6)	0.371	−1.0 (−2.2 to 0.1)	0.084
60-64	46.2 (7,051/15,273)	46.2 (6,351/13,735)	0.1 (−1.1 to 1.2)	0.901	−1.5 (−2.6 to −0.3)	0.011
65-69	40.0 (6,834/17,088)	39.5 (5,625/14,253)	−0.5 (−1.6 to 0.6)	0.342	−1.7 (−2.8 to −0.6)	0.002
70-74	34.7 (4,946/14,265)	33.7 (5,362/15,919)	−1.0 (−2.1 to 0.08)	0.071	−1.8 (−2.8 to −0.7)	0.001
Living area					
City with a population of ≥500 thousand people (government-designated city/23 cities of Tokyo)	47.8 (10,216/21,368)	46.2 (8,888/19,252)	−1.6 (−2.6 to −0.7)	<0.001	−1.9 (−2.8 to −0.9)	<0.001
City with a population of 150-499 thousand people	46.2 (14,176/30,704)	45.1 (12,349/27,410)	−1.1 (−1.9 to −0.3)	0.007	−1.7 (−2.5 to −0.9)	<0.001
City with a population of 50-149 thousand people	48.4 (13,354/27,572)	47.2 (11,424/24,193)	−1.2 (−2.1 to −0.4)	0.006	−1.8 (−2.7 to 1.0)	<0.001
City with a population of <50 thousand people	48.9 (6,482/13,252)	48.3 (6,237/12,909)	−0.6 (−1.8 to 0.6)	0.333	−1.0 (−2.2 to 0.2)	0.095
Town/village	53.1 (7,200/13,550)	51.6 (6,108/11,846)	−1.6 (−2.8 to −0.3)	0.012	−2.4 (−3.6 to −1.2)	<0.001
Educational level
Elementary/junior high school	30.0 (2,350/7,826)	28.3 (1,601/5,659)	−1.7 (−3.3 to −0.2)	0.028	−1.7 (−3.2 to −0.2)	0.031
High school	44.4 (23,214/52,282)	42.6 (18,955/44,518)	−1.8 (−2.5 to −1.2)	<0.001	−1.8 (−2.4 to −1.2)	<0.001
Vocational school/junior or technical college	54.0 (17,531/32,485)	51.6 (16,210/31,392)	−2.3 (−3.1 to −1.6)	<0.001	−1.8 (−2.6 to −1.0)	<0.001
University/graduate school	60.2 (8,333/13,853)	58.7 (8,240/14,041)	−1.5 (−2.6 to −0.3)	0.013	−1.1 (−2.2 to 0.03)	0.056
Health insurance plan						
Employee insurance (insured person)	58.5 (21,541/36,846)	58.1 (20,456/35,205)	−0.4 (−1.1 to 0.4)	0.332	−0.5 (−1.2 to 0.2)	0.197
Employee insurance (dependent person)	49.6 (15,177/30,572)	47.1 (11,921/25,289)	−2.5 (−3.3 to −1.7)	<0.001	−2.5 (−3.3 to −1.7)	<0.001
National health insurance	37.7 (14,710/39,028)	36.0 (12,629/35,116)	−1.7 (−2.4 to −1.0)	<0.001	−2.2 (−2.9 to −1.5)	<0.001

CI, confidence interval

The adjusted difference of 2017-2018 and 2020-2021 overall was −1.7% (−2.2 to −1.4). By subgroup, the adjusted difference was the largest in the 45-49 age group (−2.2% [−3.3 to −1.1]) among the age subgroups, in the town or village subgroup (−2.4% [−3.6 to −1.2]) among the living area subgroups, in the high school subgroup (−1.8% [−2.4 to −1.2]) and vocational school/junior or technical college subgroup (−1.8% [−2.6 to −1.0]) among the educational level subgroups, and the employee insurance (dependent person) subgroup (−2.5% [−3.3 to −1.7]) among the health insurance subgroups (Table 1). Among women aged 40-69 years old, the crude difference was −0.7% (−1.2 to −0.2), and the adjusted difference was −1.8% (−2.2 to −1.3).

As a sensitivity analysis, assuming that the non-responding 10,836 people in 2020-2021 were 10% more likely to participate in the breast cancer screening compared to the observed percentage (i.e., 47.1% [45,006/95,610] in 2020-2021), resulting in a participation rate of 57.1% (6,187/10,836), the simulated participating rate in 2020-2021 will be 48.1% ((45,006 + 6,187) / (95,610 + 10,836) = 51,193/106,446). However, this rate was still lower than the 2018-2019 rate of 48.3% (51,428/106,446). Conversely, if the non-responders were 10% less likely to participate, resulting in a rate of 37.1% (4,020/10,836), the projected participation rate for 2020-2021 will be 46.1% ((45,006 + 4,020) / (95,610 + 10,836) = 49,026/106,446), indicating an even greater decrease.

## Discussion

To our knowledge, this is the first nationwide study that identified changes in the breast cancer screening rates in Japan before and during the COVID-19 pandemic. The adjusted difference in the breast cancer screening participation rate between 2017-2018 and 2020-2021 for those aged 40-74 years was −1.7%. According to the CSLC survey, the breast cancer screening rate was 43.4% in 2013, 44.9% in 2016, 47.4% in 2019, and 47.4% in 2022 for those aged 40-69 years ^(13)^. Compared with the findings from international research ^(2), (3), (4), (5), (6)^, the decrease (or even stagnation) in the breast cancer screening participation rates in Japan is relatively small; however, it remains an important issue for the country. Breast cancer is the most frequently diagnosed cancer in women worldwide ^(20)^, and its early diagnosis through mammography screening is one of the important factors in reducing the breast cancer mortality ^(21), (22)^. For an organized mammography screening program to be effective, the participation rates must be high ^(23)^. Japan currently has a 60% target for its breast cancer screening participation rate. However, the decline during the COVID-19 pandemic has made this goal even more distant. It is necessary to continuously monitor trends in diagnosis, treatment, and mortality to examine the impact of the breast cancer screening rate decline in Japan.

Previous studies investigated the impact of the pandemic on breast cancer screening in Japan. One study reported that the number of people participating in the municipality-based breast cancer screenings substantially decreased in 2020, which was the first year of the pandemic ^(10)^. A self-reported questionnaire survey found that approximately one-fourth of women either canceled or postponed breast cancer screenings between April and May 2020 during the first nationwide state of emergency ^(24)^. A study utilizing a hospital-based cancer registry noted a reduction in screening-detected cases and an increase in symptom-detected cases when comparing 2020 to 2016-2019 ^(25)^. According to Abubakar et al., a self-reported questionnaire study showed an increase in the regular cancer screening participation rate during the pandemic (at 46.9%) compared with before the pandemic (at 38.2%) ^(11)^. However, this study used different definitions to measure the participation rates before and during the pandemic. Considering the same definition, the participation rate of 46.9% during the pandemic in the work of Abubakar et al. is similar to 47.1% in our study.

Regarding the breast cancer screening setting from 2017-2018 to 2020-2021, the participation rate for municipality-based screenings decreased, while that for worksite-based screenings increased. There are three main possible reasons for this change. First, there are differences in the screening recommendations that recipients receive. In the case of worksite-based screening, it is not required by law, and employers offer screening as part of their welfare system and social responsibility to employees ^(26)^. However, employers can include worksite-based screenings in their mandatory annual health checkups ^(27)^, and employers may more strongly and directly encourage employees to participate in cancer screening. Besides, municipal-based screening is available to any eligible resident, but there is no compulsion, or if there is, only a little. Some municipalities mail breast cancer screening information and coupons to residents in the relevant year, while some municipalities do not. Furthermore, the degree of recommendation by mail is lower than that of a direct recommendation from the employer. For these reasons, the decision to undergo municipal-based screening is largely left to the individual. We believe that even more so during the pandemic, some women considered cancer screening as “non-essential and non-urgent outings” and refrained from undergoing municipality-based screenings. It was not until January 2022 that the MHLW began calling for “cancer screening is an essential outing,” and no such call was made between 2020 and 2021.

Second, the Japanese government recommended extending municipality-based mass screenings during its first state of emergency declaration ^(28)^. There was no recommendation to extend during the second, third, and fourth state of emergency declarations, but the capacity for mass screening may have been reduced from the pre-pandemic levels because of the need for infection control measures.

Third, worksite-based screenings may have become widespread during this period. In 2009, a national project, called “Corporate Action to Promote Cancer Control,” was launched to improve the cancer screening rate ^(29)^. This project aimed to promote the improvement of the cancer screening participation rate in the workplace through corporate collaboration, thereby fostering a social momentum to tackle cancer positively. The goal is to achieve a cancer screening rate of 60% or more by encouraging companies to take the initiative to promote the importance of cancer screening. Approximately 5,500 companies and organizations participated in the program, which we believe led to the spread of worksite-based screenings.

We also examined the subgroups most affected by the COVID-19 pandemic. By subgroup, the largest decrease was observed in the 45-49 age group among the age subgroups, the town or village subgroup among the living area subgroups, the high school and vocational school/junior or technical college subgroup among the educational level subgroups, and the employee insurance (dependent person) subgroup among the health insurance subgroups. The possible reasons as to why they did not receive breast cancer screenings during the pandemic include the abovementioned differences in screening opportunities, personal thoughts, and financial issues. We believe it is important to follow the breast cancer screening participation rate in the future, especially for the group showing a large decline in participation rates.

This study had some limitations. First, the response rates for 2017-2018 and 2020-2021 were 72.1% and 68.0%, respectively, suggesting a decline of the survey collection. However, in our sensitivity analysis with simulation, even if the non-responding people in 2020-2021 were 10% more likely to participate in the breast cancer screening than the observed percentage among responding people, although unlikely, we found that our conclusion (i.e., decrease in the breast cancer screening participation rate from 2017-2018 to 2020-2021) will remain unchanged. Second, the data were self-reported; hence, a misclassification bias can occur, although it is likely non-differential. Finally, factors not measured by the CSLC exist, such as personal history of breast cancer, as suggested in our previous study ^(15)^.

In this study, we quantified the impact of the COVID-19 pandemic on the breast cancer screening in Japan using the CSLC data. It is important to continue to evaluate how the decreased participation rates caused by the pandemic will change in the future.

## Article Information

### Conflicts of Interest

None

### Sources of Funding

This work was supported by the MHLW Research on Emerging and Re-emerging Infectious Diseases and Immunization (Program Grant Number JPMH23HA2011 and JPMH24HA2015).

### Acknowledgement

We thank Editage (www.editage.jp) for the English language editing.

### Author Contributions

Study concept and design: CK and MI; data acquisition: RM, TK, HI, and NT; data maintenance: JK, YT, and YS; statistical data analysis: CK and MI; supervision: HI and NT; data interpretation and manuscript preparation: all authors contributed to the drafting and critical revision of the manuscript. All authors have approved the final manuscript. Hiroyasu Iso and Nanako Tamiya share last authorship.

### Approval by Institutional Review Board (IRB)

This study was approved by the Ethics Committee of the University of Tsukuba, Ibaraki, Japan (approval number 1754-1).

### Data Availability

We obtained data from the MHLW of Japan and are not allowed to share these data with other parties. Researchers who meet the criteria for access can acquire de-identified participant data from the MHLW.

### ORCID iD

Masao Iwagami: 0000-0001-7079-0640
